# The Microscope in Medicine

**Published:** 1891-02-21

**Authors:** Frank J. Wethered


### The Microscope in Medicine.

By Frank J. Wethered, M.D.

### VI.—MOUNTING SPECIMENS.

In the previous article two methods of staining were described, one by hæmatoxylin and eosin and the other by carmine and picric acid. It will be remembered that after the use of the counter-stains specimens were washed in water. The next process is that of dehydration. This is effected by means of absolute alcohol. With sections of ordinary thickness, about four or five minutes is ample time for this. As, however, the alcohol rapidly dissolves out the counter-stain, it is better to add to the spirit a few drops of a saturated alcoholic solution of eosin or picric acid, as the case may be.

The sections have now to be "cleared." The object of this is to fill the interstices of the section with a material which assimilates well with the mounting medium presently to be added. There are many "clearing reagents" in common use. The one most usually employed is oil of cloves; this, however, has some disadvantages. In the first place it has a clinging and somewhat pungent odour, and in the second place it dissolves the celloidin (if this substance has been used for embedding), and also rapidly removes eosin and picric acid. Creasote has the advantage of being cheaper, and has a less unpleasant smell, but otherwise has the same inconvenient properties as oil of cloves. Cedar oil has a more agreeable odour, does not dissolve celloidin, nor remove the counter-stains, but has a tendency to stiffen the specimens, and render their manipulation a matter of difficulty. On the whole, we consider that for ordinary work creasote is to be preferred, but it must not be used when employing aniline dyes—this point will be referred to again in another article. The creasote, then, having been placed in a shallow capsule, the sections are removed one by one from the absolute alcohol, and then carefully transferred to the oil. Caution must be used to see that the specimens are not at once completely immersed in the creasote, otherwise, owing to the rapid separation of the spirit, the sections are very apt to fly to pieces, and thus all the previous labour on them is wasted. The lifter, therefore, should be laid flat on the oil, so that the reagent may be allowed very gradually to come in contact with the specimen. The lifter is then removed from beneath the section, which should be allowed to sink, being gently depressed if necessary with the lifter.

The section will soon be seen to become quite transparent; the most convenient way of observing this is to hold the capsule over some dark object, such as the sleeve of a black coat. Should any opaque white spots be noticed, which signify that all the water has not been abstracted, the sections must be again put back in the absolute alcohol, and the process of clearing re-attempted.

When this has been successfully accomplished the section-lifter is carefully placed under one of the specimens, which is drawn on to it by means of a needle, so as to lie quite flat. The lifter must then be raised straight out of the fluid, to prevent the oil draining off, and a clean glass slide having been prepared the section is gradually tilted on to it, one corner fixed with a needle, and the lifter slowly withdrawn, when, owing to the excess of oil, the section easily floats off. Still retaining a hold of the specimen, the glass is tilted so as to allow the oil to run off, and care must again be exercised here so as to arrange the section flat on the glass. The slide is now rested against some object so as to allow the clearing reagent to drain off as much as possible. After about five minutes the slide is laid flat and cleaned around the section with a soft cloth. A drop of Canada balsam dissolved in sylol is then placed on it; a clean cover-glass is put down with one of its edges near the specimen, and then lowered on to it by means of a needle. If necessary, gentle pressure is used to expel any air bubbles, or to cause the balsam to flow more evenly. Should an empty space be left under the glass showing that sufficient balsam has not been used, another drop must be placed near the edge of the cover-glass, when the fluid will at once run in and displace the air. Should any air bubbles still remain, it is better not to make any further effort to get rid of them; they will probably be expelled by the contraction of the balsam.

The preparation must now be placed flat in a box, and protected from the dust; in about three days it will be quite dry. It should, of course, be carefully labelled with the name of the disease and organ from which it was taken, and also the method of staining and date of its completion. The above mode of mounting is the one usually employed, but it is not suitable for all cases. The most notable exception is the preparation of sections which are intended to demonstrate fatty changes. As before hinted, alcohol should altogether be avoided during the course of hardening, staining, and mounting. Such specimens should therefore be hardened in Müller's fluid, stained in hæmatoxylin and eosin, and mounted in what is known as a "fluid medium." The most useful one is glycerine. After the sections have been stained in eosin, and washed in water, they are placed directly on a slide. As much moisture as possible is then removed by means of filter papers, and a small drop of glycerine placed on the section. This should be just sufficient to occupy the space beneath the cover glass, and practice alone can enable one to judge the exact quantity. A clean cover-glass is then put on it in the same manner as was described when using balsam. After the cover-glass is laid flat, any excess of glycerine which has escaped is carefully wiped away with a cloth. It is almost useless to attempt to remove air bubbles by pressure, and their presence can only be avoided by care in lowering the glass on to the glycerine.

The glass must now be fixed. The best medium to employ for this purpose is Canada balsam sufficiently diluted. A glass rod is dipped into the balsam, and when removed allowed to drain, retaining only one or two drops. These are carefully applied to one edge of the cover-glass, when, by gently running the rod completely round the glass, sufficient balsam is left to fix the cover. Another favourite cementing material is dammar varnish. This has the following composition: Gum dammar, two parts; gum mastic, one part; turpentine, four parts; chloroform, two parts. The gums are well mixed with the turpentine and chloroform until they are dissolved, and the solution is filtered. It is used in precisely the same way as described for balsam. Whilst on the subject of mounting another preparation may here be described, which is very useful for use with fresh specimens, or those which have been stained with the aniline dyes for the demonstration of amyloid material. This is known as Farrants' solution. This consists of gum arabic, glycerine, and water, in equal parts. As, however, it is very troublesome to make, it will be found preferable to buy the solution already made. It is used in the same way as glycerine, but has the advantage of slightly drying at the edge, and so fixing the cover-glass, in fact scarcely requires any cementing material round the glass, although as a precautionary measure it is always wiser to apply a ring of Dammar varnish. Farrants' solution is less disposed to render sections too transparent, as is the case with glycerine. If circular glasses are used instead of square ones, what is known as "Shadbolt's turn table" is very convenient, as the "ringing" of the specimens may then be accurately accomplished.

It will add a finish to the appearance of the specimens if, in addition to the cement, a layer of zinc white is put as a rim round the glass. It makes the latter more secure, becoming in time as hard as enamel. Zinc white cement may be procured at any of the dealers of microscopic apparatus.

